# Evaluation of a Regional Tobacco Control Program (Greater Manchester’s *Making Smoking History*) on Quitting and Smoking in England 2014–2022: A Time-Series Analysis

**DOI:** 10.1093/ntr/ntae145

**Published:** 2024-06-08

**Authors:** Sarah E Jackson, Emma Beard, Jamie Brown

**Affiliations:** Department of Behavioural Science and Health, University College London, London, UK; SPECTRUM Consortium, Edinburgh, UK; Department of Epidemiology and Public Health, University College London, London, UK; Department of Behavioural Science and Health, University College London, London, UK; SPECTRUM Consortium, Edinburgh, UK

## Abstract

**Introduction:**

This study aimed to assess the impact of Greater Manchester’s *Making Smoking History* program—a region-wide smoking cessation programs launched in January 2018—on key smoking and quitting outcomes.

**Methods:**

Data were from a nationally representative monthly survey, 2014-2022 (*n* = 171 281). We used interrupted time-series analyses (Autoregressive Integrated Moving Average [ARIMA] and generalized additive models [GAM]) to examine regional differences between Greater Manchester and the rest of England, before and during the program’s first five years. Outcomes were rates of quit attempts and overall quits among smokers, quit success rates among smokers who tried to quit (preregistered outcomes), and current smoking prevalence among adults (unregistered outcome).

**Results:**

Results showed mixed effects of the program on quitting. Primary ARIMA models showed comparative reductions in quit success rates (change in quarterly difference between regions = –11.03%; 95% CI –18.96; –3.11) and overall quit rates in Greater Manchester compared with the rest of England (–2.56%; 95% CI –4.95; –0.18), and no significant change in the difference in the quit attempt rate (+2.95%; 95% CI –11.64; 17.54). These results were not consistently observed across sensitivity analyses or GAM analyses. Exploratory ARIMA models consistently showed smoking prevalence in Greater Manchester declined more quickly than in the rest of England following the initiation of the program (–2.14%; 95% CI –4.02; –0.27).

**Conclusions:**

The first five years of Greater Manchester’s *Making Smoking History* program did not appear to be associated with substantial increases in quitting activity. However, exploratory analyses showed a significant reduction in the regional smoking rate, over and above changes in the rest of England over the same period.

**Implications:**

Taken together, these results show a relative decline in smoking prevalence in Greater Manchester but equivocal data on quitting, introducing some uncertainty. It is possible the program has reduced smoking prevalence in the absence of any substantial change in quitting activity by changing norms around smoking and reducing uptake, or by reducing the rate of late relapse. It is also possible that an undetected effect on quitting outcomes has still contributed to the program’s impact on reducing prevalence to some degree. It will be important to evaluate the overall impact of the program over a longer timeframe.

## Introduction

Tackling smoking remains a public health priority in England^1^ but smoking prevalence and stop smoking service provision vary substantially by local authority.^2,3^ Evidence suggests regional and citywide campaigns can play a valuable role in reducing smoking prevalence over and above any national tobacco control activity.^4^

In 2017, a comprehensive regional tobacco control program was developed in Greater Manchester to reduce smoking prevalence.^5^ Titled *Making Smoking History*, the program set an ambitious aim to make Greater Manchester the first global city region to “make smoking history” by reducing smoking prevalence at an unprecedented pace and scale (Greater Manchester is a metropolitan county covering ten districts and including ~3 million people). At the time, smoking prevalence was 17.5% in Greater Manchester; approximately 17% higher than the national average for England (14.9%).^6^ The program officially launched in 2018 with a multi-pronged approach including mass-media campaigns, outdoor events throughout the summer, and localized amplification of the national campaigns (eg Stoptober). A timeline of the program’s activity to date is provided in Supplementary File 1.

Five years on from the start of the *Making Smoking History* program, smoking prevalence in Greater Manchester is at an all-time low.^7^ However, it is important to understand how changes in smoking and quitting in Greater Manchester over this period compare with other regions of England, where smoking prevalence has also fallen steadily.^8^ If the program increases quitting activity over and above what is being achieved in other regions without this type of dedicated tobacco control activity, it could provide a blueprint for similar programs in other major cities in the United Kingdom or overseas. Quantifying the added value of the program is also important in determining whether continued funding is justified.

This study aimed to evaluate the impact of the *Making Smoking History* program in Greater Manchester on rates of quit attempts and cessation among smokers, and the rate of quit success among smokers who tried to quit (collectively referred to as “quitting activity”). We also explored the program’s impact on smoking prevalence in an unplanned analysis. We did not examine the impact of the program on the uptake of smoking because we did not have sufficient numbers of participants within regional subgroups to analyze changes in smoking prevalence among young adults.

## Materials and Methods

### Preregistration

The analysis plan was preregistered on Open Science Framework (https://osf.io/3td9e/). In addition to our preplanned analyses, we included current smoking prevalence as an additional outcome and added three unplanned sensitivity analyses (described in the Statistical Analysis section). All of these unplanned analyses were conducted after we saw the results of the preplanned analyses.

### Design

Data were from the Smoking Toolkit Study (STS), a monthly cross-sectional survey of a representative sample of adults in England.^9,10^ STS uses a hybrid of random probability and simple quota sampling to select a new sample of 1700 adults representative of the adult population in England each month. Comparisons with other national surveys and sales data indicate that key sociodemographic and smoking variables are nationally representative.^9,11^

STS data were collected face-to-face up to February 2020. However, social distancing restrictions under the COVID-19 pandemic meant no data were collected in March 2020, data from April 2020 onwards were collected via telephone, and the lower age bound for participation was increased from 16 to 18 years because of changes in consenting procedures. The telephone-based data collection uses similar sampling and weighting approaches to the face-to-face interviews and the two modalities show good comparability.^12–14^

Since June 2018, an additional Greater Manchester sample has been recruited quarterly to provide greater statistical power to detect changes in smoking and quitting outcomes in this region associated with the *Making Smoking History* program. This additional data collection has been coordinated and funded by the program, independent of the main STS data collection, but uses the same questionnaire and market research company to collect the data. Before the COVID-19 pandemic, these data were collected via face-to-face (*n* ~ 360 per quarter) and telephone interviews (*n* ~ 900 in quarters 1 and 3 and *n* ~ 1200 in quarters 2 and 4). Since the COVID-19 pandemic, all additional Greater Manchester data have been collected via telephone (*n* ~ 1300 per quarter).

For the present study, we used monthly data from respondents from March 2014 (the first wave to capture data on local authority areas) to November 2022 (the latest data available at the time of analysis), including the additional Greater Manchester sample from June 2018 to September 2022. To match the age range of participants recruited since the pandemic, we excluded those aged 16 and 17 surveyed before March 2020.

### Measures

#### Exposure

The region in England was categorized based on participants’ local authority area. For our primary analysis, the region was dichotomized to Greater Manchester versus other (ie the rest of England). However, there are other areas of England with regional tobacco control programs (eg “Fresh” in the North East; freshne.com), so the comparison with the rest of England may underestimate the true impact of the *Making Smoking History* program in Greater Manchester. We therefore ran a sensitivity analysis comparing Greater Manchester with the Sheffield City Region, which was selected as a matched control region with the same prevalence of smoking in 2017 as Greater Manchester (17.7%, compared with 17.5% in Greater Manchester) and a similar trend since 2018,^6^ but no comparable regional tobacco control program.

#### Outcomes

Outcomes were the monthly prevalence of: (i) quit attempts among smokers, (ii) quit success rates among those who tried, (iii) the overall quit rate among smokers, and (iv) current smoking among adults. The first three outcomes were assessed via two questions asked of past-year smokers in each STS wave:

1. How many serious attempts to stop smoking have you made in the last 12 months?
2. How long did your most recent serious quit attempt last before you went back to smoking?

The prevalence of *quit attempts* in each month was calculated as the number of respondents who reported having made one or more quit attempts in the past 12 months divided by the number of past-year smokers.

The *success rate of quit attempts* in each month was calculated as the number of respondents reporting that they were “still not smoking” (in response to the second question) divided by the number who reported having made a quit attempt.

The *overall quit rate* was calculated as the number of respondents reporting that they were still not smoking divided by the number of past-year smokers.

*Smoking prevalence* was calculated as the number of respondents who reported being a current smoker divided by the number of adults surveyed.

#### Covariates

Covariates for the individual-level analyses included participants’ age, gender, and social grade (ABC1, which includes managerial, professional, and intermediate occupations, versus C2DE, which includes small employers and own‐account workers, lower supervisory and technical occupations, and semi‐routine and routine occupations, state pension, never worked and long‐term unemployed).

### Statistical Analysis

Our primary analyses used representative data from the main STS sample (ie excluding the additional Greater Manchester sample), weighted to match the population in England.^9^

Our primary analysis used Autoregressive Integrated Moving Average (ARIMA) models to estimate the impact of the intervention at the population level.^15–17^ Data were aggregated quarterly. The independent variable was coded 0 up until but not including January 2018 (the launch of the *Making Smoking History* program in Greater Manchester) and then 1 until November 2022 inclusive, reflecting the first five years of the program. Dependent variables were regional differences in the prevalence of each outcome between Greater Manchester (intervention region) and the rest of England (control region). A planned sensitivity analysis compared differences between Greater Manchester and the Sheffield City Region (matched control region). Where any prevalence values were zero (*n* = 17 data points) or implausibly high (>80%, *n* = 3 data points), values were imputed using Kalman smoothing for univariate time-series data.^18^ Results are reported with and without imputation. Standard recommended procedures^15,19–21^ were used to select the ARIMA models (see Supplementary File 2 for details). The *B* coefficients from these analyses can be interpreted as the total change in the series mean from the pre to the postintervention period attributable, in the absence of confounding, to the intervention.

A secondary segmented regression analysis was then conducted using Generalized Additive Models (GAMs) at the individual, non-aggregated level, modeling the difference in changes over time between people living in Greater Manchester and those living in the control regions. These models allow the fitting of seasonal smoothing terms and therefore allow seasonality to be taken into account. The GAM model unadjusted for sex, age, and social grade was specified as: $y_{i}=\beta_{0}+\beta_{1}trend_{i}+\beta_{2}level_{i}+\beta_{3}slope_{i}+\beta_{4}trend_{i}*region_{i}+$$\beta_{5}level_{i}*region_{i}+\beta_{6}slope_{i}*region_{i}+\beta_{7}seasonality_{i}+e_{i}$ where trend is a variable coded 1 … *n* (*n* is the total number of quarters to the end of the series) reflecting the trend over time; level is a dummy variable coded 0 before the intervention and 1 after to reflect a step-level change; and slope is a variable coded 0 before the start of the program and 1 … *n* after and reflects the change in the preintervention slope postintervention. Seasonality was modeled using a smoothing term, with survey month coded 0 = January through 11 = December and cyclic cubic splines specified. We also ran an adjusted model for each outcome, including age, gender, and social grade as covariates.

Our rationale for using both ARIMA and GAM models was to triangulate across approaches that each depend on different assumptions and offer distinct benefits and limitations.^22^ ARIMA is a “true” time-series approach that can take account of autocorrelation by the inclusion of seasonal and non-seasonal autoregressive and moving average terms.^21^ It therefore allows underlying trends to be accurately accounted for. It examines population-level effects and can therefore offer insight into whether the intervention works at the population level (which will be important for informing decisions on continued funding and expansion to other regions). As a population-level analysis, it is robust to individual-level confounding. However, ARIMA cannot directly test interactions—in this case, the interaction between time (pre vs. postintervention) and region (exposed vs. unexposed)—which is why we modeled the difference in our outcomes between regions. It is also limited by the possibility of population-level confounding, such as the introduction of other population-level initiatives (eg policy) and changes in the demographic profiles of the population. GAM examines individual-level effects, so it is subject to individual-level—but not population-level—confounding. It allows for differences between regions to be tested directly using an interaction term and has greater statistical power to detect significant associations. In addition, while ARIMA removes the underlying trend, GAM models it. However, GAM relies on the assumption that trends in the preintervention period were the same across regions, which can be problematic when sample sizes are relatively small (resulting in volatile data). The two approaches produce parameter estimates with qualitatively different interpretations.

We repeated the ARIMA models in three unplanned sensitivity analyses. The first coded the start of the intervention as July 2017 (when the *Making Smoking History* program was first published), rather than January 2018, because visual inspection of the data indicated a rise in quit attempts in Greater Manchester before the program officially launched. The second modeled changes in the absolute prevalence of each outcome in Greater Manchester, without adjustment for prevalence in a control region, to explore the impact of the control groups on the pattern of results. The third added participants from the additional Greater Manchester sample commissioned by the program to the main STS sample, with data for Greater Manchester reweighted to match the population in Greater Manchester (rather than using the standard England weights), to explore differences in the pattern of results when all available data from participants in Greater Manchester were included. This additional sample was only available post-intervention and used a different weighting approach to the main STS sample, so we chose not to include it in the primary analyses to ensure consistency across the time series.

To provide more insight into changes in quitting activity in Greater Manchester since the *Making Smoking History* program started in 2018, we used individual-level data from all Greater Manchester participants (in the main STS and additional samples) to examine trends in quit attempts, quit success rates, overall quit rates, and smoking prevalence within the intervention region since March 2018. These results are presented and discussed in Supplementary File 3.

## Results

Data were collected from 171 281 adults aged ≥ 18 years in the core Smoking Toolkit Study sample between March 2014 and November 2022, of whom 31 878 were past-year smokers. The analyzed sample included 7036 adults in Greater Manchester (1648 past-year smokers; current smoking prevalence in Greater Manchester 21.8%, 95% CI = 20.7 to 22.8) and 164 245 in the rest of England (30 230 past-year smokers; current smoking prevalence in the rest of England 17.4%, 95% CI = 17.2 to 17.6), of whom 4327 were in the Sheffield City Region (893 past-year smokers; current smoking prevalence in Sheffield 19.0%, 95% CI = 17.7 to 20.2). Weighted sociodemographic characteristics (overall and in the first and last years of the study period) are provided in Supplementary File 4.

**Figure 1.**
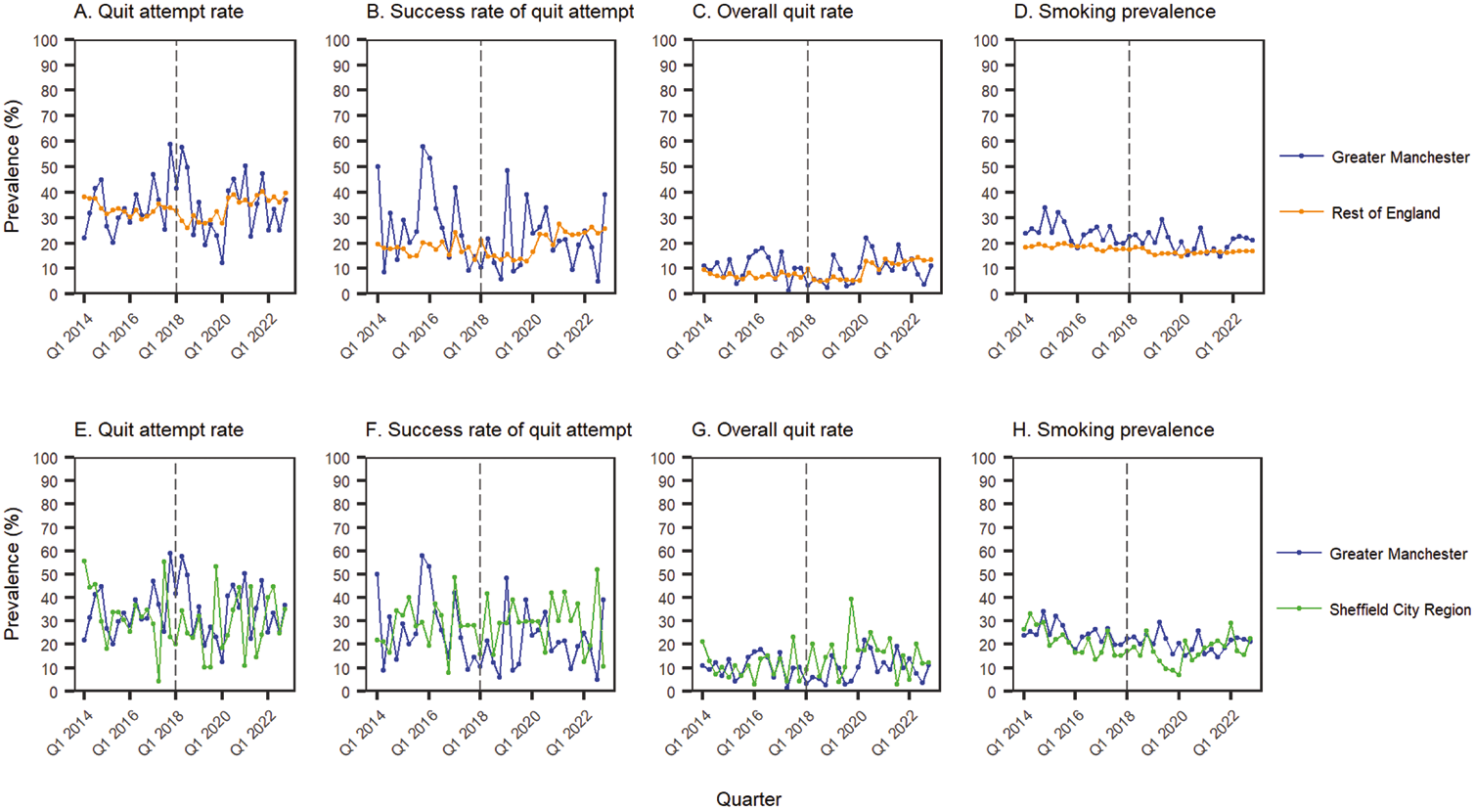
Quarterly prevalence of quit attempts, quit success, overall quits, and current smoking in Greater Manchester compared with the rest of England and the Sheffield City Region, March 2014–November 2022. The vertical gray line indicates the timing of the start of the intervention. For Greater Manchester and Sheffield City Region, the prevalence of quit success and overall quits in some months was implausibly low (zero) or high (>80%) and so values were imputed using Kalman smoothing for univariate time-series data.^18^

Figure 1 shows the quarterly prevalence of quit attempts, quit success among those who made an attempt, the overall quit rate among past-year smokers, and smoking prevalence among adults in Greater Manchester compared with the rest of England and Sheffield.

### Population-Level Analysis (ARIMA Models)

Table 1 summarizes the results of the ARIMA models. Regional differences in the weighted prevalence of quit attempts, the success rate of quit attempts, the overall quit rate, and smoking prevalence over the time series are shown in Supplementary File 5.

**Table 1.** Results of the ARIMA models assessing the association between the implementation of the intervention and prevalence of quit attempts, quit success, overall quits, and current smoking

	*B*	95% CI	*p*
**Mean difference in the prevalence of quit attempts**			
Model 1 (Greater Manchester – the rest of England)	2.95	–11.64, 17.54	.692
Model 2 (Greater Manchester – Sheffield City Region)	5.04	–6.86, 16.95	.406
**Mean difference in the success rate of quit attempts**			
Model 3 (Greater Manchester minus the rest of England)			
* Imputation*	–9.53	–18.07, –0.98	.029
* No imputation*	–9.29	–18.44, –0.13	.047
Model 4 (Greater Manchester minus Sheffield City Region)			
* Imputation*	–8.30	–16.09, –0.50	.037
* No imputation*	–7.19	–20.60, 6.23	.294
**Mean difference in the overall quit rate**			
Model 5 (Greater Manchester minus the rest of England)			
* Imputation*	–3.25	–6.42, –0.07	.045
* No imputation*	–4.05	–6.18, –1.92	<.001
Model 6 (Greater Manchester minus Sheffield City Region)			
* Imputation*	–4.48	–6.52, –2.43	<.001
* No imputation*	–2.67	–7.15, 1.81	.242
**Mean difference in smoking prevalence**			
Model 7 (Greater Manchester minus the rest of England)	–2.14	–4.02, –0.27	.025
Model 8 (Greater Manchester minus Sheffield City Region)	0.74	–4.97, 6.46	.799

Model 1 (0,1,1)(1,0,0)_4_ MA1 *p* < .001, SAR1 *p* = .016; Model 2 (0,1,1) MA1 *p* < .001; Model 3 imputation (0,0,0), no imputation (0,0,0); Model 4 imputation (0,0,0), no imputation (0,0,0); Model 5 imputation (0,0,0), no imputation (0,0,0)(0,0,1)_4_ SMA1 *p* = .004; Model 6 imputation (0,0,0)(0,0,2)_4_ SMA1 *p* = .986, SMA2 *p* = .017, no imputation (0,0,0); Model 7 (0,0,0)(1,0,0)_4_ SAR1 *p* = .029; Model 8 (0,1,1)(2,0,0)_4_ MA1 *p* < .001, SAR1 *p* = .003, SAR2 *p* < .001.

There was no significant change in the mean quarterly difference in the prevalence of quit attempts among smokers in Greater Manchester compared with the rest of England (Figure 1, A) or compared with Sheffield (Figure 1, E).

There was a significant reduction in the mean quarterly difference in the quit success rate in Greater Manchester compared with the rest of England, from 10.30% points in the preintervention period to 0.78 in the postintervention period (Figure 1, B), and compared with Sheffield (Figure 1, F), from –0.09 to –8.39. The latter result was not statistically significant in the model without imputation.

There was also a significant reduction in the mean quarterly difference in the overall quit rate in Greater Manchester compared with the rest of England, from 3.37% points in the preintervention period to 0.12 in the postintervention period (Figure 1, C), and compared with Sheffield, from 0.02 to –4.46, respectively (Figure 1, G). The latter result was not statistically significant in the model without imputation.

In contrast, there was a significant reduction in the mean quarterly difference in smoking prevalence in Greater Manchester compared with the rest of England (Figure 1, D), from 5.99% points in the preintervention period to 3.85 in the postintervention period. There was no significant change compared with Sheffield (Figure 1, H).

### Sensitivity Analyses

A similar pattern of results was found when the timing of the start of the intervention was modeled as July 2017 rather than January 2018, to account for increases in quitting activity in the lead-up to the program launch (see Supplementary File 6).

No evidence of an increase (or significant decrease) in quitting outcomes was detected when changes in absolute prevalence in Greater Manchester were modeled without adjustment for prevalence in other regions (Supplementary File 7). However, a significant step-level fall in smoking prevalence was detected (*B* = –3.92, 95% CI –6.50, –1.34, *p* = .003), from a mean of 24.50% in the preintervention period to 20.58% in the postintervention period.

When additional participants recruited as part of the additional Greater Manchester sample between June 2018 and September 2022 were included in the analysis, and data for Greater Manchester were reweighted to match the population in Greater Manchester, we detected significant increases in the mean quarterly difference in prevalence of quit attempts (*B* = 8.05, 95% CI 3.15, 12.95, *p* = .001) and quit success (*B* = 6.02, 95% CI 0.80, 11.25, *p* = .024) in Greater Manchester compared with the rest of England (Supplementary File 8). There was no significant difference in the mean quarterly difference in the overall quit rate or smoking prevalence, although the point estimate for the latter was similar to our primary analysis (*B* = –2.11 vs. –2.14).

### Individual-Level Analysis (GAMs)

**Figure 2.**
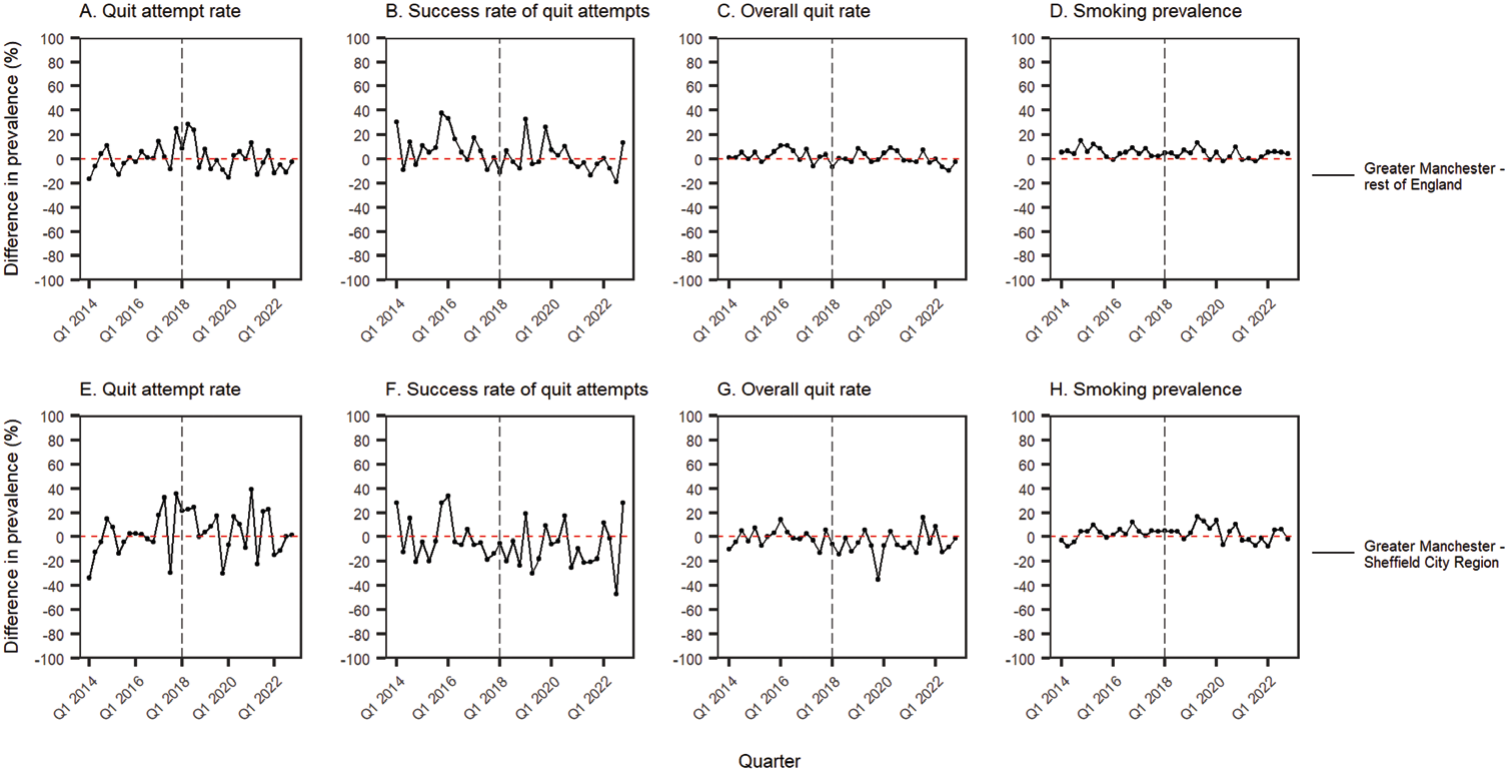
Modeled prevalence of quit attempts, quit success, overall quits, and current smoking in Greater Manchester compared with the rest of England and the Sheffield City Region, March 2014–November 2022. The vertical gray line indicates the timing of the start of the intervention. Lines represent weighted prevalence modeled using GAMs, adjusted for seasonality, age, gender, and social grade (ie, the adjusted line of best fit). Points represent unadjusted weighted quarterly prevalence (ie, raw values, as shown in Figure 1).

Table 2 and Figure 2 summarize the results of the GAM analysis.

**Table 2.** Results of the GAMs assessing the association between the implementation of the intervention and quit attempts, quit success, cessation, and current smoking

	Greater Manchester vs. rest of England						Greater Manchester vs. Sheffield City Region					
	Unadjusted			Adjusted			Unadjusted			Adjusted		
	RR	95% CI	*p*	RR	95% CI	*p*	RR	95% CI	*p*	RR	95% CI	*p*
**Quit attempts** ^a^												
Trend	0.99	0.99, 1.00	.016	0.99	0.99, 1.00	.010	0.96	0.93, 0.99	.023	0.96	0.93, 0.99	.018
Level	0.88	0.82, 0.94	<.001	0.88	0.83, 0.94	<.001	1.02	0.65, 1.61	.919	1.05	0.67, 1.65	.819
Slope	1.03	1.02, 1.03	<.001	1.03	1.02, 1.03	<.001	1.06	1.02, 1.10	.006	1.06	1.02, 1.10	.004
Region	0.80	0.62, 1.03	.086	0.75	0.59, 0.97	.028	0.66	0.46, 0.93	.017	0.63	0.45, 0.88	.007
Trend × region	1.03	1.01, 1.06	.014	1.03	1.01, 1.06	.010	1.06	1.02, 1.11	002	1.07	1.02, 1.11	.002
Level × region	1.13	0.86, 1.49	.380	1.11	0.85, 1.46	.438	0.97	0.58, 1.64	.922	0.93	0.55, 1.55	.777
Slope × region	0.94	0.91, 0.97	<.001	0.94	0.92, 0.97	<.001	0.91	0.87, 0.96	<.001	0.91	0.87, 0.96	<.001
**Quit success** ^b^												
Trend	1.00	0.99, 1.01	.902	1.00	0.98, 1.01	.864	0.98	0.92, 1.05	.563	0.98	0.92, 1.05	.592
Level	0.79	0.66, 0.95	.012	0.81	0.67, 0.96	.018	1.45	0.59, 3.57	.417	1.42	0.57, 3.51	.450
Slope	1.03	1.02, 1.05	<.001	1.03	1.02, 1.05	<.001	1.01	0.93, 1.10	.741	1.02	0.94, 1.10	.701
Region	1.77	1.08, 2.91	.024	1.66	1.01, 2.74	.047	1.14	0.57, 2.27	.716	1.00	0.50, 1.99	.992
Trend × region	0.98	0.93, 1.03	.344	0.98	0.93, 1.03	.421	0.99	0.91, 1.08	.810	1.00	0.92, 1.09	.973
Level × region	1.10	0.56, 2.16	.786	1.11	0.57, 2.19	.758	0.66	0.22, 1.97	.456	0.66	0.22, 1.97	.453
Slope × region	1.00	0.94, 1.07	.966	1.00	0.94, 1.07	.988	1.02	0.92, 1.13	.680	1.01	0.91, 1.12	.806
**Cessation (overall quits)** ^a^												
Trend	1.00	0.99, 1.01	.901	1.00	0.98, 1.01	.770	0.98	0.92, 1.05	.642	0.98	0.92, 1.05	.596
Level	0.75	0.63, 0.88	<.001	0.76	0.64, 0.89	<.001	1.43	0.63, 3.28	.393	1.52	0.67, 3.49	.319
Slope	1.06	1.04, 1.07	<.001	1.06	1.04, 1.07	<.001	1.01	0.93, 1.10	.768	1.02	0.94, 1.10	.689
Region	1.49	0.90, 2.49	.125	1.34	0.80, 2.25	.262	1.00	0.48, 2.08	.992	0.88	0.42, 1.84	.738
Trend × region	1.00	0.95, 1.05	.929	1.00	0.95, 1.05	.998	1.01	0.93, 1.10	.827	1.01	0.93, 1.10	.743
Level × region	0.73	0.37, 1.44	.361	0.77	0.39, 1.52	.446	0.39	0.14, 1.12	.080	0.40	0.14, 1.13	.083
Slope × region	0.99	0.93, 1.06	.805	0.99	0.93, 1.06	.810	1.04	0.94, 1.15	.469	1.03	0.93, 1.14	.534
**Current smoking** ^c^												
Trend	0.99	0.99, 1.00	<.001	0.99	0.99, 1.00	<.001	0.95	0.93, 0.97	<.001	0.95	0.94, 0.97	<.001
Level	0.95	0.91, 0.99	.022	0.97	0.93, 1.01	.102	1.01	0.77, 1.32	.954	0.99	0.77, 1.29	.966
Slope	1.00	1.00, 1.01	.038	1.01	1.00, 1.01	.012	1.07	1.04, 1.10	<.001	1.06	1.04, 1.09	<.001
Region	1.40	1.20, 1.64	<.001	1.23	1.07, 1.43	.005	0.87	0.69, 1.09	.215	0.78	0.63, 0.96	.022
Trend × region	0.99	0.98, 1.01	.289	1.00	0.99, 1.01	.973	1.04	1.01, 1.06	.008	1.04	1.01, 1.06	.002
Level × region	1.07	0.89, 1.29	.490	1.02	0.85, 1.22	.830	1.01	0.73, 1.40	.929	1.00	0.74, 1.37	.976
Slope × region	1.00	0.98, 1.02	.834	1.00	0.98, 1.01	.604	0.94	0.91, 0.97	<.001	0.94	0.92, 0.97	<.001

Trend is the underlying trend prior to the intervention. The slope is the change in the underlying trend associated with the intervention.

All results are adjusted for seasonality. Adjusted models are additionally adjusted for age, gender, and social grade.

^a^Among past-year smokers.

^b^Among past-year smokers who made a quit attempt.

^c^Among adults.

Assuming the underlying trend was modeled correctly, the results showed no significant interaction between region and a step-level change in quit attempts, quit success, cessation (ie overall quits), or current smoking following initiation of the intervention.

A significant interaction between region and slope (ie change in trend) was identified for quit attempts (Figure 2, A and E). Stratified models revealed that before the intervention, the prevalence of quit attempts increased by 3% per quarter in Greater Manchester (RR_trend_ = 1.03, 95% CI 1.00 to 1.05); after the start of the intervention, this rate of increase stalled (RR_slope_ = 0.97, 95% CI 0.94 to 0.99), reducing to 0% per quarter (RR_trend_ × RR_slope_ = 1.03 × 0.97 = 1.00). By contrast, quit attempts had been falling by 1% per quarter before the intervention in the rest of England (RR_trend_ = 0.99, 95% CI 0.99 to 1.00) and by 4% per quarter in Sheffield (RR_trend_ = 0.96, 95% CI 0.93 to 0.99). After the start of the intervention, these trends reversed (rest of England RR_slope_ = 1.03, 95% CI 1.02 to 1.03; Sheffield RR_slope_ = 1.06, 95% CI 1.02 to 1.11), increasing by 2% per quarter in both the rest of England and Sheffield specifically.

A significant interaction between region and slope was also identified for current smoking, in the comparison with Sheffield (Figure 2, H). Stratified models revealed that before the intervention, smoking prevalence fell by 1% per quarter in Greater Manchester (RR_trend_ = 0.99, 95% CI 0.98 to 1.00) and by 4% per quarter in Sheffield (RR_trend_ = 0.96, 95% CI 0.94 to 0.98). After the start of the intervention, the rate of decline did not change significantly in Greater Manchester (RR_slope_ = 1.00, 95% CI 0.99 to 1.02, *p* = .707). However, the declining trend reversed in Sheffield (RR_slope_ = 1.06, 95% CI 1.03 to 1.09, *p* < .001), with smoking prevalence increasing by 2% per quarter. There was no significant difference in the change in trend between Greater Manchester and the rest of England (Figure 2, D).

The interaction between region and slope for quit success and cessation in Greater Manchester was not significant for the rest of England (Figure 2, B and C) or Sheffield (Figure 2, F and G).

## Discussion

Our primary analyses indicated the first five years of the *Making Smoking History* program were not associated with a substantial increase in the regional quit attempt rate, success rate of quit attempts, or overall quit rate among smokers. The population-level (ARIMA) analysis showed no significant change in quit attempts in Greater Manchester relative to the rest of England. However, the individual-level (GAM) analysis indicated that the rising trend in quit attempts observed preintervention may have stalled during the intervention. The population-level analysis showed a significant *fall* in the success of quit attempts and overall quit rate in Greater Manchester relative to the rest of England. This was not consistently observed in the individual-level analysis, which showed no significant interaction between region and a step-level change or change in trend, nor in an unplanned sensitivity analysis that included participants in the additional Greater Manchester sample, which showed a significant increase in rates of quit attempts and quit success relative to the rest of England. Results were similar when Greater Manchester was compared with the Sheffield City Region, a matched control region with no regional tobacco control program.

However, our results provide some evidence for the benefit of the program on smoking prevalence. The population-level analysis showed a greater reduction in regional smoking prevalence in Greater Manchester than in the rest of England. Before the program, smoking prevalence was 5.99% points higher in Greater Manchester than in the rest of England; after the program launched this difference fell to 3.85% points. The individual-level analysis indicated trends in smoking prevalence differed between Greater Manchester and Sheffield following the launch of the program: smoking prevalence continued to decline steadily in Greater Manchester while the (more rapid) preprogram rate of decline in Sheffield reversed and smoking prevalence began to increase in this region. As a matched control, Sheffield serves as a counterfactual: what might have occurred in Greater Manchester had the *Making Smoking History* program not been implemented. While one might usually expect a continuous trend in the absence of any intervention, this is not necessarily likely to be the case in the context of the COVID-19 pandemic, which occurred during the intervention period and was associated with substantial changes in our outcomes of interest.^12^ This underscores the importance of comparing changes in the target region with those that occurred elsewhere in England over the same period.

Differences between the population-level and individual-level results can be explained by the differences in modelling approaches. The ARIMA models analyzed step-level changes in quitting outcomes at the population level, and focused on the differences between regions, whereas the GAMs modeled step-level changes and changes in trends at the individual level, and modeled the separate trends before and after the change in each region as well as their interaction. Using the two different approaches offers value in terms of triangulation, as consistent results across methodologies are less likely to be artifacts.^22^ Differences between the primary ARIMAs and the sensitivity analysis including the additional Greater Manchester participants may be attributable to the inclusion of additional participants post but not preintervention, or the different weighting approach.

Considering our results collectively, seeing a fall in smoking prevalence in Greater Manchester with equivocal data on quitting (ie different patterns of results across sensitivity analyses) is somewhat surprising, and introduces some uncertainty to our results. Other regional and national programs aiming to promote smoking cessation in England and overseas have tended to show benefits in terms of increased rates of quit attempts and cessation.^23–27^ There may be several possible explanations for the pattern of results we observed. First, the program may have had an impact on smoking prevalence in the absence of any substantial change in quitting activity by changing norms around smoking and reducing uptake, or by reducing the rate of late relapse (ie ≥1 year post-quitting). Secondly, an undetected effect on quitting outcomes may still be contributing to the program’s impact on reducing prevalence to some degree. Thirdly, the greater decline in smoking prevalence in Greater Manchester may have been unrelated to the program and, for example, may have instead reflected changing population cohorts or demographics in the region. Across the study period, a number of tobacco control policies were introduced,^28^ although these were at a national level so would not be expected to have a greater impact in Greater Manchester than elsewhere in England.

There are several factors that may have affected our ability to detect true effects on quitting outcomes, had there been any: (i) Greater Manchester appeared to be outperforming the rest of England prior to the start of the intervention, with higher rates of quit success and overall quits; (ii) the COVID-19 pandemic was associated with a substantial increase in quitting,^12,13^ which may have differentially impacted any effects of these types of regional programs by driving up quit rates in control regions in the absence of typical intervention; (iii) Greater Manchester experienced greater adverse impacts of the COVID-19 pandemic (including extensive regional lockdowns and higher rates of mortality than the rest of England^29^) which may have suppressed the impact of the program; and (iv) the program has provided national leadership, which has influenced national interventions and policy in England, and may thereby have indirectly improved the “control” regions. In addition, some tobacco control interventions, for example changing the age of sale from 16 to 18,^30^ have had longer-term impacts on trends by changing norms and culture, and it will be important to evaluate the overall impact of the program over a longer timeframe. Nonetheless, the smoking prevalence results suggest the program may have had a positive impact on the proportion of adults who smoke, possibly mitigating against a slowing or even reversal of the steady decline over time in the absence of any intervention (as observed in Sheffield).

Strengths of this study include the large, representative sample of adult smokers across all regions of England and monthly data collection. In addition, the modeling approaches offered insight into changes in Greater Manchester over and above changes that occurred elsewhere in England over the same period. There were also limitations. First, sample sizes for Greater Manchester and Sheffield were small, introducing substantial volatility to the time series. As ARIMA models are sensitive to outliers and large shifts in the data, this could have affected the results. However, we ran models with and without imputation of extreme values and found similar results. Secondly, the preintervention period was relatively short. Combined with the volatility of the data for Greater Manchester and Sheffield, this made it difficult to be sure if we accurately modeled preintervention trends in our outcomes in the GAM models. We observed that the differences between regions in the preintervention trends were not constant over time (Figure 2), potentially violating the parallel trends assumption. This limitation should be considered when interpreting the individual-level results. Thirdly, the analyses of the smoking prevalence outcome were unregistered, and so this part of the results should be considered exploratory and replicated in future long-term evaluations of the program. Smoking prevalence outcomes are more distal, and thus usually less sensitive, than proximal quitting outcomes (and the reason we preregistered quitting outcomes for a program focused on quitting). It is unexpected to detect changes in smoking prevalence but not quitting, yet we discuss the possible reasons above (changes to norms, uptake, or long-term relapse) and these should be further examined in future work. Fourthly, while we compared changes over time in Greater Manchester with two control regions that offered different advantages, neither provided a perfect control: the rest of England provided a large sample size but was not matched on smoking prevalence or demographic profile and included other regions with dedicated local tobacco control activity, while Sheffield provided a better (although still imperfect) match but had a limited sample size. In addition, the Sheffield City Region was selected as a control region with no large-scale dedicated regional tobacco campaign, but we became aware of substantial local tobacco control activity in Sheffield after running our analyses. Comparisons with Sheffield may therefore underestimate the impact of Greater Manchester’s program. Nonetheless, when we modeled changes over time in Greater Manchester without adjustment for a control region (ie, prevalence in Greater Manchester, rather than the difference in prevalence between regions), we did not find an association between the start of the intervention and an increase in any quitting outcome, indicating the limitations of the control groups were not the reason we did not detect a rise in quit rates. Fifthly, the measure of exposure (lives in Greater Manchester vs. lives elsewhere) may not accurately distinguish between participants who were and were not exposed to the campaign. People traveling to Greater Manchester from neighboring regions (eg for work) may have contaminated the control group. If exposure to the campaign encouraged people living in other regions to make a quit attempt or access local Stop Smoking Services, then this contamination would have limited the statistical power to detect what may have been meaningful increases in success rates. Sixthly, we used a hybrid sampling approach rather than random probability sampling. However, comparisons with other sources suggest the survey recruits a nationally representative sample and produces similar estimates of key smoking variables.^9,11^ Seventhly, the outcome measures relied on quit attempts in the last year rather than the last month, which meant the assessment period after the intervention would not have related exclusively to the intervention. ARIMA analyses are designed to model such noise. Finally, while the individual-level models were adjusted for age, gender, and socioeconomic position, there may be residual confounding by unmeasured variables (eg ethnicity).

In conclusion, the first five years of Greater Manchester’s *Making Smoking History* program did not appear to be associated with substantial increases in quitting activity. However, exploratory analyses showed a significant reduction in the regional smoking rate, over and above changes in the rest of England over the same period.

## Supplementary material

Supplementary material is available at *Nicotine and Tobacco Research* online.

ntae145_suppl_Supplementary_Data_S1

ntae145_suppl_Supplementary_Data_S2

ntae145_suppl_Supplementary_Data_S3

ntae145_suppl_Supplementary_Data_S4

ntae145_suppl_Supplementary_Data_S5

ntae145_suppl_Supplementary_Data_S6

ntae145_suppl_Supplementary_Data_S7

ntae145_suppl_Supplementary_Data_S8

## Data Availability

Data used for these analyses are available on Open Science Framework (https://osf.io/3td9e/).
