# Supplementary material for: Evaluation of a Regional Tobacco Control Program (Greater Manchester’s Making Smoking History) on Quitting and Smoking in England 2014–2022: A Time-Series Analysis
Source: Nicotine Tob Res. 2024 Jun 8;26(12):1728–36. doi: 10.1093/ntr/ntae145 (PMC11581995; doi:10.1093/ntr/ntae145)
Supplement: ntae145_suppl_Supplementary_Data_S1 [file ntae145_suppl_supplementary_data_s1.docx]

**Supplementary File 1:** Greater Manchester’s Making Smoking History activity timeline

**Table.** Greater Manchester’s Making Smoking History activity timeline

| **Activity** | **Dates** | **Notes** |
| --- | --- | --- |
| Publication of GM’s Making Smoking History Programme | July 2017 | https://www.gmhsc.org.uk/wp-content/uploads/2018/04/Tobacco-Free-Greater-Manchester-Strategy.pdf |
| Insight into Stop Smoking Campaigns | Summer 2017 | Decision to commission and deliver the Don’t be the One campaign in early 2018 |
| Boost to Stoptober | September – October 2017 |  |
| Smoke Free Pregnancy programme System Engagement and launch across GM | December 2017 |  |
| Commissioning National Smoking Helpline for GM launch on original My City Health GM website (no longer in commission) | January 2018 |  |
| CRUK E-Cigarette campaign in GM | January 2018 | CRUK’s first ever mass media campaign on e-cigarettes was delivered in GM. CRUK’s £200k investment in the campaign saw in a significant increase in smokers attempting to quit (40% to 53%) and trying an e-cigarette (42% to 52%) suggesting a positive impact on quitting behaviour. |
| Official Programme launch by GM Mayor | February 2018 |  |
| Don’t Be The One Mass Media Campaign Launch | February 2018 | Don’t Be the 1 integrated multi-media intervention. The TV advert running across ITV reached 69% of routine and manual worker (C2DE) audience on average 16 times. The campaign exceeded its target that 90,000 (just over 20%) smokers would take some quit related action because of the campaign; around one third of GM smokers, 134,000 people actively engaged in quitting |
| History Makers Public Conversation | March – May 2018 | Engagement with 7500 GM residents with overwhelming support for the programme |
| Salford Swap to Stop Vaping pilot | January – March 2018 | The early 2018 Salford Swap to Stop pilot enabled 1000 smokers, living in social housing to access free e-cigarette starter kits, alongside local stop smoking support. 60% of those receiving the initial stop smoking intervention continued to engage with local stop smoking support and 63% achieved a carbon monoxide (CO) validated 4 week quit |
| Smokefree Pregnancy Programme phase one launch | February – March 2018 | First phase implementation |
| GM boost to Smoking Toolkit Study commissioned to deliver quarterly data | June 2018 |  |
| Smokefree Summer- smokefree outdoor events across GM | June – August 2018 | Programme supported existing local events to be smokefree with toolkit/stand/promotional staff |
| GM wide Smokers Insight research and segmentation project completed | August 2018 | Segmented GM smokers in 8 typologies- insight used to create new GM campaign and stop smoking brand- launched Jan 2019 |
| CURE secondary care tobacco dependency treatment programme 6 month pilot launched at Wythenshawe Hospital site | October 2018 | https://thecureproject.co.uk/ |
| GM amplification of National Stoptober campaign | September – October 2018 |  |
| Launched GM’s strategic Tackling Illicit Tobacco framework | December 2018 |  |
| NHS Long Term Plan Published- GM’s CURE Inpatient Treating Tobacco Dependency and Smokefree Pregnancy programmes both cited as exemplar best practice | January 2019 |  |
| Launch of new GM You Can branding and ‘Exsmoker’ campaign | March – May 2019 | Based on in-depth insight and segmentation of smokers in GM |
| Commenced Smokefree Pregnancy Programme RCT- 3 arm-trial for use of incentives up to 12 months post-partum | February 2019 |  |
| Commissioned LGBT to fund a part time LGBT Making Smoking History Project Lead to deliver specific projects addressing LGBT smoking rates | March 2019 |  |
| Commissioned 3rd sector infrastructure organisations with £100,000 grant to work with community organisation to support a Social Movement of History makers and Engagement community members with a focus on smokefree spaces | Summer 2019 | Covid had a significant impact on the delivery of this project |
| First burst of Keep it Out illicit tobacco demand reduction campaign | May 2019 | The first burst of the campaign in May 2019 lead to large seizures - 637,000 illicit cigarettes and over 150kg of illicit hand rolling tobacco |
| North of England 16 Cancers Campaign- Amplified in GM | June 2019 |  |
| Launched primary care e-learning modules on Gateway-C GP e-learning platform | August 2019 | These have not really been utilised due to lack of capacity to promote and then impact of covid on primary care |
| GM Pride events supported | Summer 2019 |  |
| GM Stoptober amplification | September – October 2019 |  |
| Final phase of Smokefree Pregnancy Programme implemented |  | Apart from Wigan |
| Always on digital/social between campaign bursts | September 2020 |  |
| New Year Ex-Smoker Campaign | January 2020 |  |
| No Smoking Day campaign activity through HITs radio- boost to national No Smoking Day activity | March 2020 |  |
| Covid – first lock down | March 2020 |  |
| All Stop Smoking Services moved to digital/telephone support including Smokefree Pregnancy Programme | March 2020 |  |
| Launch of Smoke Free App for GM residents- initially a response to Covid | April 2020 | Promoted through quit for covid communications activity |
| Humanitarian response to Covid- swap to stop vaping project for homeless | April – December 2020 | Vaping starter kits and app support provided to those housed in temporary accommodation |
| National Quit for Covid Campaign- Amplified in GM | May – June 2020 |  |
| Don’t Wait Campaign | July 2020 |  |
| Publication of WHO Panorama Case Study on the programme | June 2020 | https://apps.who.int/iris/handle/10665/332671 |
| CURE Acute Tobacco Dependency Treatment Programme rolled out to a further hospital sites | August – October 2020 | All sites were live by March 2020 Wigan, Bury, Oldham, Stockport, Tameside, Salford |
| Keep It Out Campaign | November 2020 |  |
| Smokefree Pregnancy programme launched in Wigan | January 2021 |  |
| Don’t Wait Campaign | January – February 2021 |  |
| Digital Solution for Smokefree Pregnancy Data implemented | April – October 2021 |  |
| Smokefree Pregnancy vaping pilot at Manchester Foundation Trust | September 2021 – January 2022 |  |
| Smokefree Pregnancy Programme risk perception training | September – December 2021 |  |
| Last recruitment for Smokefree Pregnancy RCT | August 2021 |  |
| Evaluation phase for humanitarian response | November 2020 – July 2021 |  |
| Launch and delivery of new Make Smoking History Brand and Never Quit Quitting GM Stop Smoking Campaign | August – September 2021 | Smoke Free App offer included through campaigns |
| Stockport Substance Misuse Vaping Pilot | Autumn 2021 |  |
| CURE Pharmacy discharge pilot launch | September 2020 |  |
| Keep it Out Campaign Illicit Tobacco Campaign | November 2021 |  |
| Supported Pride Festivals | May – August 2021 | Some digital, some face to face- more light touch limited interactions |
| Digital Never Quit Quitting Campaign | December 2021 |  |
| OHID Better Health amplification | January 2022 |  |
| Never Quit Quitting Mass Media Campaign Burst | February – March 2022 | As part of no smoking day activity |
| Commenced NHS England Staff Stop Smoking app offer with fulfilment offer | March 2022 |  |
| Commenced Social Housing and Smoking Insight project | June 2022 |  |
| Commenced Smokefree outdoor spaces community consultation | August 2022 | Part of GM joining the global Partnerships for Healthy Cities- Grant of $100,000 received to deliver a project on Smokefree spaces |
| CURE rollout to remaining sites not delivering (Bolton and remaining Manchester Foundation Trust sites) | Soft launch October 2022 |  |
| Khan Review on Smokefree 2030 published. Never Quit Quitting Campaign and GMs CURE inpatient and Smokefree Pregnancy programmes all cited as exemplar best practice | June 2022 |  |
| Selected as MH early implementor site | July 2022 |  |
| Never Quit Quitting Campaign | August 2022 |  |
| Wigan CURE Vaping Pilot launched | August 2022 |  |
| Pride Festival activity | May – September 2022 | Smokefree spaces/events promotion and communications/messaging |
| NHS England Staff offer rollout to Northern Care Alliance Trust | September 2022 |  |
| Stoptober Amplification | September – October 2022 |  |
| Local Maternity and Neonatal System Equality and Equity Audit around Smokefree Pregnancy Programme | May – September 2022 |  |
| ARC CURE Long Term Plan Evaluation of Implementation | April 2020 – ongoing |  |
