# Supplementary material for: Evaluation of a Regional Tobacco Control Program (Greater Manchester’s Making Smoking History) on Quitting and Smoking in England 2014–2022: A Time-Series Analysis
Source: Nicotine Tob Res. 2024 Jun 8;26(12):1728–36. doi: 10.1093/ntr/ntae145 (PMC11581995; doi:10.1093/ntr/ntae145)
Supplement: ntae145_suppl_Supplementary_Data_S2 [file ntae145_suppl_supplementary_data_s2.docx]

**Supplementary File 2:** Additional information on ARIMA model selection

First, each time-series was assessed for outlying values that may bias the results using a procedure described in Chen and Liu (*tsoutliers* function in the *forecast* package).^1^ One outlier was identified for the difference in overall quit rate between Greater Manchester and Sheffield City Region (imputed); it was replaced with the recommended value (*tsclean* function). Secondly, the plots of the differenced data and unit root tests (i.e. Osborn-Chui-Smith-Birchenhall test and Kwiatkowski, Phillips, Schmidt, and Shin test) were used to determine the number of seasonal and non-seasonal differences required for the time-series to be stationary.^2,3^ Thirdly, we assessed the autocorrelation function (ACF) and partial autocorrelation function (PACF) and used an automated algorithm (*auto.arima()* in the *forecast* package) to identify the ARIMA model terms.^4^ This algorithm iteratively searches over a series of potential ARIMA models for the one with the lowest AIC or BIC, with several constraints applied to avoid convergence problems (including setting the maximum value of p and q to 5 and P and Q to 2)^4^ Finally, the Ljung-Box test for white noise and residual plots of the best fitting models were checked for additional correlation (thus the need for additional MA/AR seasonal or non-seasonal terms) and the coefficients of the correlation terms assessed for significance and whether they fell within the bounds of stationarity and invertibility.^5,6^

**References**

1. López-de-Lacalle, J. *tsoutliers R Package for Detection of Outliers in Time Series*. https://cran.r-project.org/web/packages/tsoutliers/tsoutliers.pdf (2022).

2. Lee, D. & Schmidt, P. On the power of the KPSS test of stationarity against fractionally-integrated alternatives. *J. Econom.* **73**, 285–302 (1996).

3. Osborn, D. R. Seasonality and the Order of Integration for Consumption. *Oxf. Bull. Econ. Stat.* **50**, 361–377 (1988).

4. Hyndman, R. J. & Khandakar, Y. Automatic Time Series Forecasting: The forecast Package for R. *J. Stat. Softw.* **27**, 1–22 (2008).

5. Yaffee, R. A. & McGee, M. *An Introduction to Time Series Analysis and Forecasting: With Applications of SAS® and SPSS®*. (Academic Press, 2000).

6. Yaffee, R. A. *An Introduction to Forecasting Time Series with Stata*. (Taylor and Francis, 2012).
