# Supplementary material for: Evaluation of a Regional Tobacco Control Program (Greater Manchester’s Making Smoking History) on Quitting and Smoking in England 2014–2022: A Time-Series Analysis
Source: Nicotine Tob Res. 2024 Jun 8;26(12):1728–36. doi: 10.1093/ntr/ntae145 (PMC11581995; doi:10.1093/ntr/ntae145)
Supplement: ntae145_suppl_Supplementary_Data_S3 [file ntae145_suppl_supplementary_data_s3.docx]

**Supplementary File 3:** Trends in quitting activity and smoking prevalence within Greater Manchester since the start of the programme, by social grade

**Rationale**

Reducing inequalities in smoking^1,2^ has been a strong focus of the programme, with activities targeting high-risk groups including a Smokefree Pregnancy Programme, a ‘Swap to Stop’ vaping pilot for people living in social housing, and appointment of an LGBT project lead to deliver projects to reduce smoking among the LGBT community (e.g., at pride events). The programme has continued to provide targeted regional tobacco control activity since its launch.

Establishing the extent to which any impacts of the programme on quitting activity and smoking prevalence differ across socioeconomic groups is important for understanding the programme’s impact on inequalities and targeting resources going forward.

**Methods**

To provide more insight into changes in quitting activity and smoking prevalence in Greater Manchester since the *Making Smoking History* programme started in 2018, we used individual-level data from all Greater Manchester participants (in the main STS and additional samples) to examine trends in quit attempts, quit success rates, overall quit rates, and smoking prevalence within the intervention region since March 2018.

For each outcome, we used log-binomial regression to test the association with time (survey quarter), adjusting for age, gender, and social grade. Time was modelled using restricted cubic splines with three knots placed at the earliest, middle, and latest quarters, to allow relationships with time to be flexible and non-linear, while avoiding categorisation. To explore moderation by social grade, we repeated the relevant models including the interaction between social grade and quarter – thus allowing for time trends to differ across sub-groups (i.e., social grades C2DE vs. ABC1).

**Results**

An additional sample of 18,243 adults (2,497 past-year smokers) in Greater Manchester was recruited between June 2018 and September 2022. Combined with the 5,414 adults (1,147 past-year smokers) in Greater Manchester recruited as part of the main STS sample over this period, this provided a total sample within Greater Manchester of 23,657 adults (3,644 past-year smokers).

In this sample, the weighted prevalence of quit attempts over this period was 39.2% [95%CI 37.2-41.2], the success rate of quit attempts was 26.4% [23.4-29.4], and the overall quit rate was 12.3% [11.0-13.6]. These rates did not change significantly from June 2018 to September 2022 (**Figure A-C**). However, smoking prevalence fell significantly (*p*=0.004) from 15.6% [14.3-17.2] in Q3-2018 to 12.8% [12.1-13.6] in Q3-2020, then rose to 15.2% [13.8-16.7] by Q3-2022 (modelled estimates; **Figure D**).

Time trends differed significantly by social grade for the success rate of quit attempts (**Figure F;** *p*_interaction_=0.006), the overall quit rate (**Figure G**; *p*_interaction_=0.008), and smoking prevalence (**Figure H**; *p*_interaction_=0.034). Among smokers from more advantaged social grades (ABC1), there was a significant increase in quitting between Q3-2018 and Q4-2020 (the overall quit rate rose from 7.8% [95%CI 5.3-11.5%] to 19.0% [15.7-22.9%]) followed by an uncertain decline (to 13.7% [10.0-18.6%] in Q3-2022); while among less advantaged social grades (C2DE), there was no significant change over time (11.3% [10.0-18.6%] and 9.5% [6.6-13.8%] in Q3-2018 and Q3-2022, respectively). A similar pattern was observed for quit attempts (**Figure D**), although the interaction was not statistically significant. Likewise, among adults from more advantaged social grades (ABC1), there was a significant decline in smoking prevalence between Q3-2018 and Q4-2020 (from 13.4% [11.8-15.2] to 9.2% [8.4-10.1]) followed by a rise (to 11.7% [10.2-13.4] in Q3-2022); while among less advantaged social grades (C2DE), there was no significant change over time (19.5% [17.3-22.0] and 19.9% [17.7-22.5] in Q3-2018 and Q3-2022, respectively).


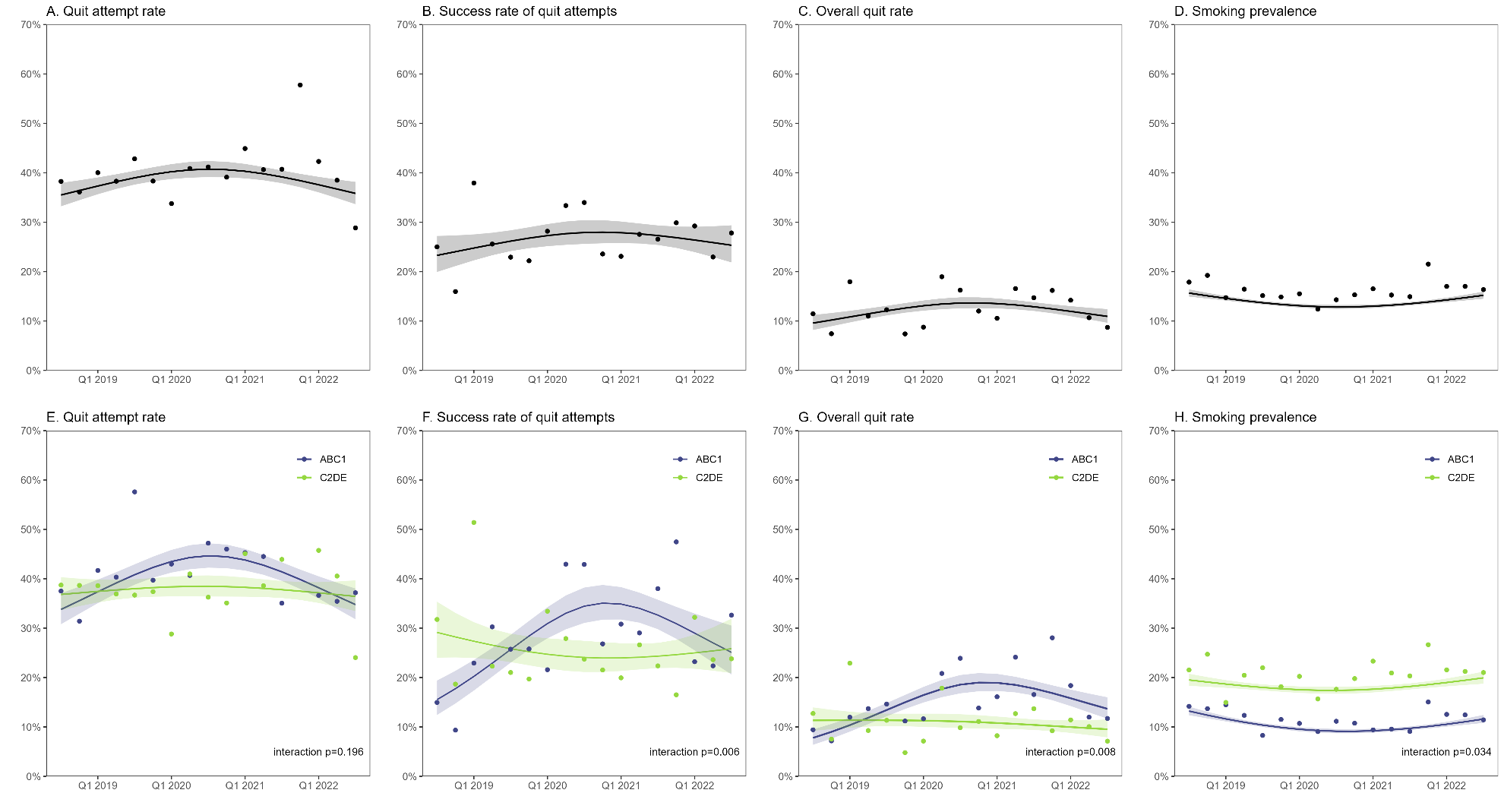


##### Figure. Time trends in the prevalence of quit attempts, quit success, overall quits, and current smoking among smokers in Greater Manchester since the start of the *Making Smoking History* programme, June 2018 to September 2022. Data are presented overall (panels A-D) and by occupational social grade (panels E-H). Lines represent modelled weighted prevalence by survey quarter, modelled non-linearly using restricted cubic splines (three knots), adjusting for covariates. Shaded bands represent standard errors. Points represent unadjusted weighted prevalence by quarter. ABC1 = more advantaged social grades; C2DE = less advantaged social grades.

**Discussion**

Within Greater Manchester, there was some evidence of an increase in quitting among smokers from more advantaged social grades during the first three years of the programme, with no significant change among those from less advantaged social grades. Changes in smoking prevalence mirrored this pattern, with a decline observed among more but not less advantaged social grades. Although there was no control group for this analysis – and as such, it is not clear how far these differences mirror what was happening in other regions of England over the same period – these results suggest any benefit of the programme may have been concentrated among more advantaged smokers.

The *Making Smoking History* programme has targeted high-risk groups including pregnant women, social housing residents, and members of the LGBT community, with the aim of reducing inequalities in smoking. Our results do not offer any insight into the effectiveness of these targeted activities (because sample sizes would have been too small to detect changes in these groups).

That we did not find a particular benefit for disadvantaged smokers more broadly is consistent with other tobacco control interventions which, with the exception of price/taxation measures, generally do not show an equity-positive impact.^3,4^ However, studies suggest targeted cessation support can reduce inequalities by achieving higher reach among disadvantaged smokers to compensate for lower quit rates.^3,4^ Broadening the scope of targeted activities from the relatively small high-risk groups currently prioritised to the much larger group of socioeconomically disadvantaged smokers could improve the *Making Smoking History* programme’s equity impact. In addition, it would be helpful for researchers in the future to conduct a process evaluation to identify which parts of the programme were perceived to be more helpful and acceptable by smokers and ex-smokers living in Greater Manchester.

**References**

1. Hiscock, R., Bauld, L., Amos, A., Fidler, J. A. & Munafò, M. Socioeconomic status and smoking: a review. *Ann. N. Y. Acad. Sci.* **1248**, 107–123 (2012).

2. Hiscock, R., Dobbie, F. & Bauld, L. Smoking Cessation and Socioeconomic Status: An Update of Existing Evidence from a National Evaluation of English Stop Smoking Services. *BioMed Res. Int.* **2015**, 274056 (2015).

3. Smith, C. E., Hill, S. E. & Amos, A. Impact of population tobacco control interventions on socioeconomic inequalities in smoking: a systematic review and appraisal of future research directions. *Tob. Control* **30**, e87–e95 (2021).

4. Brown, T., Platt, S. & Amos, A. Equity impact of population-level interventions and policies to reduce smoking in adults: A systematic review. *Drug Alcohol Depend.* **138**, 7–16 (2014).
