# Supplementary material for: Evaluation of a Regional Tobacco Control Program (Greater Manchester’s Making Smoking History) on Quitting and Smoking in England 2014–2022: A Time-Series Analysis
Source: Nicotine Tob Res. 2024 Jun 8;26(12):1728–36. doi: 10.1093/ntr/ntae145 (PMC11581995; doi:10.1093/ntr/ntae145)
Supplement: ntae145_suppl_Supplementary_Data_S4 [file ntae145_suppl_supplementary_data_s4.docx]

**Supplementary File 4:** Weighted sociodemographic characteristics

**Table.** Weighted sociodemographic characteristics of participants in Greater Manchester, the rest of England, and the Sheffield City Region, overall and in the first and last years of the study

|  | **Greater Manchester** | | |  | **Rest of England** | | | |  | | **Sheffield City Region** | | | |
| --- | --- | --- | --- | --- | --- | --- | --- | --- | --- | --- | --- | --- | --- | --- |
|  | **Overall** | **2014** | **2022** |  | **Overall** | **2014** | **2022** |  | | **Overall** | | **2014** | **2022** |  |
| Age, mean (95%CI) | 46.1  (45.6-46.5) | 42.4  (40.8-44.1) | 47.2  (45.8-48.7) |  | 48.0  (47.9-48.1) | 47.6  (47.3-47.9) | 48.3  (48.0-48.6) |  | | 47.9  (47.3-48.4) | | 46.7  (45.2-48.2) | 46.8  (44.8-48.7) |  |
| Women, % (95%CI) | 51.2  (50.0-52.5) | 51.3  (46.8-55.7) | 48.5  (44.5-52.5) |  | 51.0  (50.7-51.2) | 51.0  (50.1-51.8) | 51.2  (50.3-52.0) |  | | 50.2  (48.6-51.8) | | 50.8  (46.1-55.5) | 49.2  (43.5-54.9) |  |
| Less advantaged social grades (C2DE), % (95%CI) | 45.7  (44.4-47.0) | 52.1  (47.7-56.6) | 44.0  (40.0-48.0) |  | 44.6  (44.3-44.8) | 45.2  (44.4-46.0) | 43.7  (42.9-44.6) |  | | 45.3  (43.7-46.9) | | 47.6  (42.9-52.2) | 53.9  (48.4-59.4) |  |

CI, confidence interval.
