# Supplementary material for: Evaluation of a Regional Tobacco Control Program (Greater Manchester’s Making Smoking History) on Quitting and Smoking in England 2014–2022: A Time-Series Analysis
Source: Nicotine Tob Res. 2024 Jun 8;26(12):1728–36. doi: 10.1093/ntr/ntae145 (PMC11581995; doi:10.1093/ntr/ntae145)
Supplement: ntae145_suppl_Supplementary_Data_S5 [file ntae145_suppl_supplementary_data_s5.docx]

**Supplementary File 5:** Regional differences in the weighted prevalence of quit attempts, the success rate of quit attempts, the overall quit rate, and smoking prevalence over the time-series


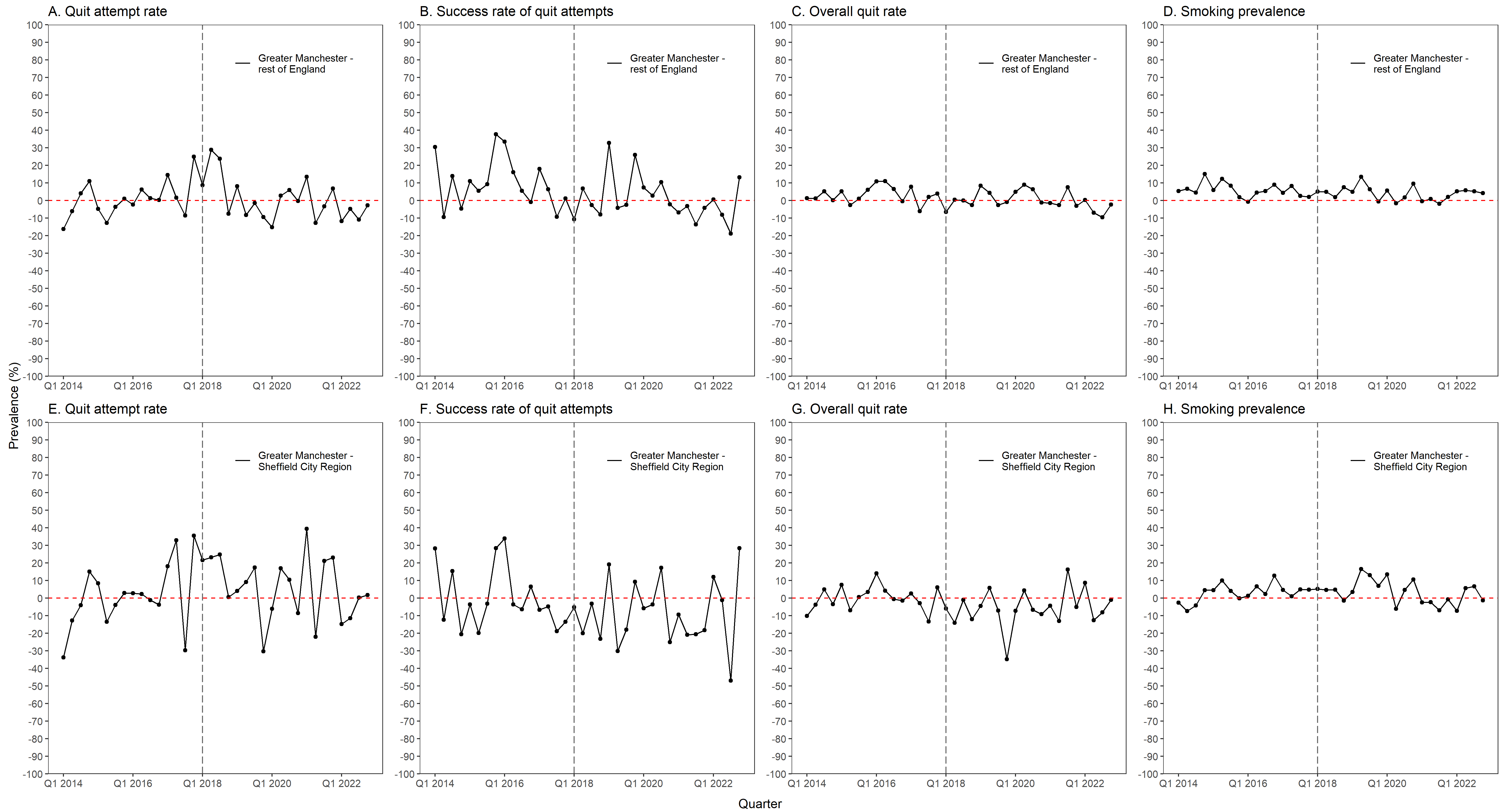


**Figure. Quarterly prevalence of quit attempts, quit success, overall quits, and current smoking in Greater Manchester compared with the rest of England and the Sheffield City Region, March 2014 to November 2022.** The vertical grey line indicates the timing of the start of the intervention. Values above 0 (indicated by the horizontal red line) indicate higher levels of quitting activity in Greater Manchester compared with the control region and values below 0 indicate lower levels. For Greater Manchester and Sheffield City Region, prevalence of quit success and overall quits in some months was zero and so values were imputed using Kalman smoothing for univariate time-series data.
