# Supplementary material for: Evaluation of a Regional Tobacco Control Program (Greater Manchester’s Making Smoking History) on Quitting and Smoking in England 2014–2022: A Time-Series Analysis
Source: Nicotine Tob Res. 2024 Jun 8;26(12):1728–36. doi: 10.1093/ntr/ntae145 (PMC11581995; doi:10.1093/ntr/ntae145)
Supplement: ntae145_suppl_Supplementary_Data_S6 [file ntae145_suppl_supplementary_data_s6.docx]

**Supplementary File 6:** Unplanned sensitivity analysis 1 – ARIMA models based on an intervention start date of July 2017

| **Table.** Sensitivity analysis: ARIMA models based on an intervention start date of July 2017 | | | |
| --- | --- | --- | --- |
|  | **B** | **95% CI** | ***p*** |
| **Mean difference in prevalence of quit attempts** |  |  |  |
| Model 1 (Greater Manchester minus the rest of England) | 0.77 | -16.24, 17.77 | 0.930 |
| Model 2 (Greater Manchester minus Sheffield City Region) | 5.05 | -7.09, 17.19 | 0.415 |
|  |  |  |  |
| **Mean difference in success rate of quit attempts** |  |  |  |
| Model 3 (Greater Manchester minus the rest of England) |  |  |  |
| *Imputation* | -12.01 | -20.40, -3.62 | 0.005 |
| *No imputation* | -12.30 | -19.31, -5.28 | 0.001 |
| Model 4 (Greater Manchester minus Sheffield City Region) |  |  |  |
| *Imputation* | -9.51 | -12.32, -6.69 | <0.001 |
| *No imputation* | -5.45 | -18.32, 7.42 | 0.406 |
|  |  |  |  |
| **Mean difference in overall quit rate** |  |  |  |
| Model 5 (Greater Manchester minus the rest of England) |  |  |  |
| *Imputation* | -3.00 | -5.40, -0.61 | 0.014 |
| *No imputation* | -4.04 | -6.24, -1.84 | <0.001 |
| Model 6 (Greater Manchester minus Sheffield City Region) |  |  |  |
| *Imputation* | -4.14 | -5.12, -3.16 | <0.001 |
| *No imputation* | -2.93 | -4.46, -1.39 | <0.001 |
|  |  |  |  |
| **Mean difference in smoking prevalence** |  |  |  |
| Model 7 (Greater Manchester minus the rest of England) | -2.54 | -4.42, -0.66 | 0.008 |
| Model 8 (Greater Manchester minus Sheffield City Region) | 0.85 | -4.96, 6.67 | 0.773 |
| Note: Model 1 (0,1,1)(1,0,0)_4_ MA1 p=0.002, SAR1 p=0.021; Model 2 (0,1,1), MA1 p<0.001; Model 3 imputation (0,0,0), no imputation (0,0,0)(1,0,0)_4_ SAR1 p=0.049; Model 4 imputation (0,0,1)(1,0,0)_4_ MA1 p=0.010, SAR1 p=0.072, no imputation (0,0,0); Model 5 imputation (0,0,0)(0,0,1)_4_ SMA1 p=0.032, no imputation (0,0,0)(0,0,1)_4_ SMA1 p=0.003; Model 6 imputation (0,0,1)(0,0,2)_4_ MA1 p=0.062, SMA1 p=0.337, SMA2 p=0.015, no imputation (0,0,1)(0,0,2)_4_ MA1 p=0.033, SMA1 p=0.511, SMA2 p=0.022; Model 7 (0,0,0)(1,0,0)_4_ SAR1 p=0.030; Model 8 (0,1,1)(2,0,0)_4_ MA1 p<0.001, SAR1 p=0.003, SAR2 p<0.001. | | | |
