# Supplementary material for: Evaluation of a Regional Tobacco Control Program (Greater Manchester’s Making Smoking History) on Quitting and Smoking in England 2014–2022: A Time-Series Analysis
Source: Nicotine Tob Res. 2024 Jun 8;26(12):1728–36. doi: 10.1093/ntr/ntae145 (PMC11581995; doi:10.1093/ntr/ntae145)
Supplement: ntae145_suppl_Supplementary_Data_S7 [file ntae145_suppl_supplementary_data_s7.docx]

**Supplementary File 7:** Unplanned sensitivity analysis 2 – ARIMA models based on absolute prevalence in Greater Manchester

| **Table.** Sensitivity analysis: ARIMA models based on absolute prevalence in Greater Manchester (i.e., no adjustment for prevalence in a control region) | | | |
| --- | --- | --- | --- |
|  | **B** | **95% CI** | ***p*** |
| **Prevalence of quit attempts** | 0.45 | -5.09, 5.99 | 0.872 |
|  |  |  |  |
| **Success rate of quit attempts** |  |  |  |
| *Imputation* | -7.41 | -16.13, 1.30 | 0.096 |
| *No imputation* | -7.17 | -16.64, 2.29 | 0.137 |
|  |  |  |  |
| **Overall quit rate** |  |  |  |
| *Imputation* | -0.90 | -4.31, 2.51 | 0.605 |
| *No imputation* | -1.90 | -5.60, 1.80 | 0.313 |
|  |  |  |  |
| **Smoking prevalence** | -3.92 | -6.50, -1.34 | 0.003 |
| Note: Quit attempts (0,0,0)(1,0,0)_4_ SAR1 p=0.045; success rate of quit attempts imputation (0,0,0), no imputation (0,0,0); overall quit rate imputation (0,0,0), no imputation (0,0,0); smoking prevalence (0,0,0). | | | |
