# Supplementary material for: Evaluation of a Regional Tobacco Control Program (Greater Manchester’s Making Smoking History) on Quitting and Smoking in England 2014–2022: A Time-Series Analysis
Source: Nicotine Tob Res. 2024 Jun 8;26(12):1728–36. doi: 10.1093/ntr/ntae145 (PMC11581995; doi:10.1093/ntr/ntae145)
Supplement: ntae145_suppl_Supplementary_Data_S8 [file ntae145_suppl_supplementary_data_s8.docx]

**Supplementary File 8:** Unplanned sensitivity analysis 3 – ARIMA models including all available participants surveyed in Greater Manchester

| **Table.** Sensitivity analysis: ARIMA models including participants in the Greater Manchester boost sample between June 2018 and September 2022, with Greater Manchester data weighted to match the population in Greater Manchester | | | |
| --- | --- | --- | --- |
|  | **B** | **95% CI** | ***p*** |
| **Mean difference in prevalence of quit attempts** |  |  |  |
| Model 1 (Greater Manchester minus the rest of England) | 8.05 | 3.15, 12.95 | 0.001 |
| Model 2 (Greater Manchester minus Sheffield City Region) | 7.82 | -5.15, 20.79 | 0.237 |
|  |  |  |  |
| **Mean difference in success rate of quit attempts** |  |  |  |
| Model 3 (Greater Manchester minus the rest of England) |  |  |  |
| *Imputation* | 6.02 | 0.80, 11.25 | 0.024 |
| *No imputation* | 6.02 | 0.72, 11.33 | 0.026 |
| Model 4 (Greater Manchester minus Sheffield City Region) |  |  |  |
| *Imputation* | 1.22 | -7.49, 9.94 | 0.783 |
| *No imputation* | -1.90 | -16.92, 13.13 | 0.804 |
|  |  |  |  |
| **Mean difference in overall quit rate** |  |  |  |
| Model 5 (Greater Manchester minus the rest of England) |  |  |  |
| *Imputation* | 1.97 | -1.52, 5.46 | 0.268 |
| *No imputation* | 1.97 | -1.52, 5.46 | 0.268 |
| Model 6 (Greater Manchester minus Sheffield City Region) |  |  |  |
| *Imputation* | -3.97 | -8.07, 0.13 | 0.058 |
| *No imputation* | -0.47 | -5.05, 4.12 | 0.842 |
|  |  |  |  |
| **Mean difference in smoking prevalence** |  |  |  |
| Model 7 (Greater Manchester minus the rest of England) | -2.11 | -4.44, 0.22 | 0.076 |
| Model 8 (Greater Manchester minus Sheffield City Region) | -1.33 | -8.58, 5.93 | 0.720 |
| Note: Model 1 (0,0,0); Model 2 (0,1,3) MA1 p<0.001, MA2 p=0.779, MA3 p=0.051; Model 3 imputation (0,0,0), no imputation (0,0,0); Model 4 imputation (0,0,0), no imputation (0,0,0); Model 5 imputation (0,0,1) MA1 p=0.023, no imputation (0,0,1) MA1 p=0.023; Model 6 imputation (0,0,0), no imputation (0,0,0); Model 7 (0,0,0); Model 8 (0,1,1)(2,0,0)_4_ MA1 p<0.001, SAR1 p=0.295, SAR2 p<0.001. | | | |

Notes: One outlier replaced in Model 3, no imputation (first data point corrected upwards); one outlier replaced in Model 5, imputation and no imputation (first data point corrected downwards).


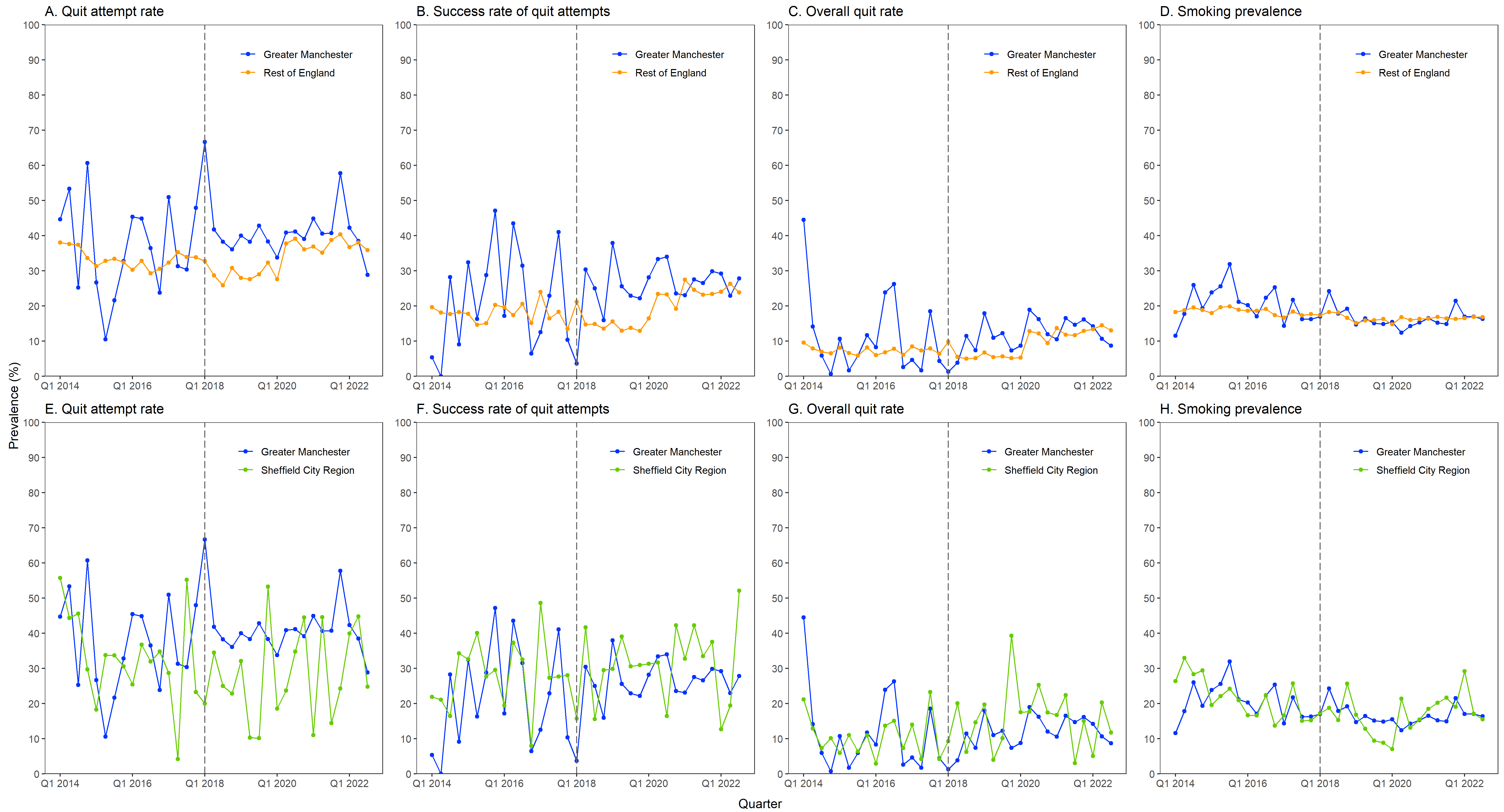


**Figure 1. Quarterly prevalence of quit attempts, quit success, overall quits, and current smoking in Greater Manchester compared with the rest of England and the Sheffield City Region, March 2014 to November 2022.** The vertical grey line indicates the timing of the start of the intervention. For Greater Manchester and Sheffield City Region, prevalence of quit success and overall quits in some months was implausibly low (zero) or high (>80%) and so values were imputed using Kalman smoothing for univariate time-series data.^25^


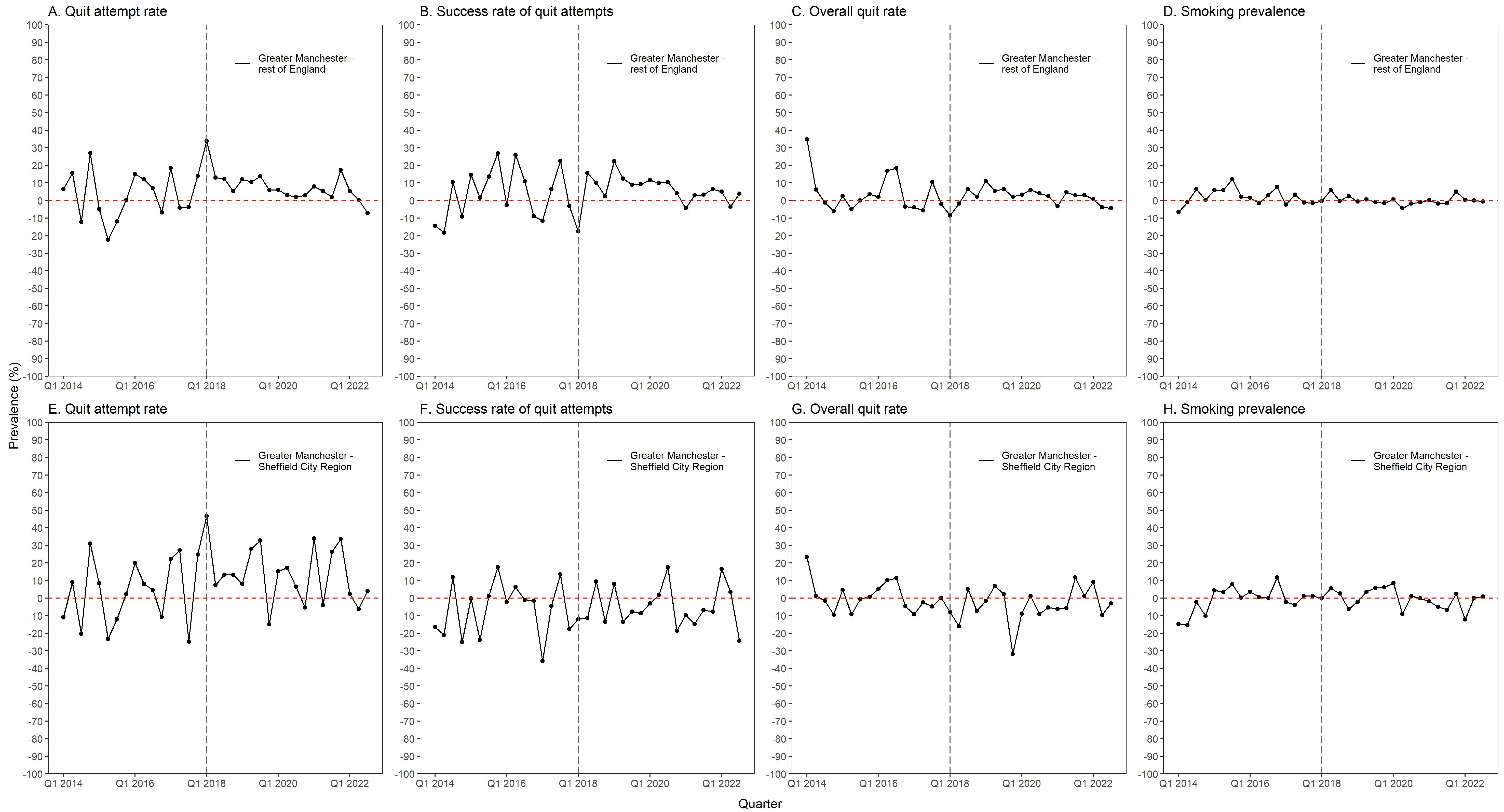


**Figure 2. Quarterly prevalence of quit attempts, quit success, overall quits, and current smoking in Greater Manchester compared with the rest of England and the Sheffield City Region, March 2014 to November 2022.** The vertical grey line indicates the timing of the start of the intervention. Values above 0 (indicated by the horizontal red line) indicate higher levels of quitting activity in Greater Manchester compared with the control region and values below 0 indicate lower levels. For Greater Manchester and Sheffield City Region, prevalence of quit success and overall quits in some months was zero and so values were imputed using Kalman smoothing for univariate time-series data.^25^
